# Short-term efficacy and adverse effects of collagenase clostridium histolyticum injections, percutaneous needle fasciotomy and limited fasciectomy in the treatment of Dupuytren’s contracture: a network meta-analysis of randomized controlled trials

**DOI:** 10.1186/s12891-022-05894-6

**Published:** 2022-10-28

**Authors:** Doha Obed, Mustafa Salim, Frederik Schlottmann, Alperen S. Bingoel, Adriana C. Panayi, Khaled Dastagir, Peter M. Vogt, Soeren Koenneker

**Affiliations:** 1 Department of Plastic, Aesthetic, Hand and Reconstructive Surgery, Hannover Medical School, Hannover, Germany; 2 Department of Human Genetics, Hannover Medical School, Hannover, Germany; 3 Division of Plastic Surgery, Department of Surgery, Brigham and Women’s Hospital and Harvard Medical School, Boston, MA USA

**Keywords:** Dupuytren contracture, Limited fasciectomy, Percutaneous needle fasciotomy, Collagenase clostridium histolyticum, Collagenase injection, Randomized controlled trial, Meta-analysis

## Abstract

**Aims:**

Dupuytren’s contracture (DC) is a chronic debilitating fibroproliferative disorder.

Common treatment options include collagenase clostridium histolyticum injections (CI), percutaneous needle fasciotomy (NF) and limited fasciectomy (LF). Superiority of one specific treatment remains controversial. This study aims to assess the short-term efficacy and safety of CI, NF, and LF for the treatment of DC.

**Methods:**

We included randomized controlled trials of CI compared with placebo, NF and LF for patients with DC. PubMed, Embase and the Cochrane Library were searched from inception to August 2021. Contracture reduction rates in treated joints (within 0–5° of full extension within 30 days), relative reduction in total passive extension deficit (TPED), occurrence of one or more adverse events and number of treatment-related adverse events per patient were the outcomes of interest. The Cochrane risk-of-bias tool was employed for quality assessment of the studies. A network meta-analysis was performed using MetaXL.

**Results:**

Nine studies met our inclusion criteria (*n* = 903). Overall, risk bias was mixed and mostly low. Short term TPED reduction achieved with LF was superior compared to CI and NF. Although CI achieved greater TPED reduction compared to NF, it was associated with the highest risk of overall adverse effects. The analyzed data was limited to a maximum three-year follow-up period and therefore insufficient for long-term outcome evaluation.

**Conclusions:**

In DC, LF may be able to provide patients with severe disease, superior flexion contracture release postoperatively. CI is a valid treatment alternative to NF, however the higher risk of overall adverse effects must be considered. The quality-of-evidence is limited due to short-term follow-up periods and a lack of standardized definitions of complications and adverse events.

## Introduction

Dupuytren’s contracture (DC) is a benign fibroproliferative disorder that results in the formation of collagen knots and fibers and ultimately in contracture of the palmar fascia. It most commonly affects the ring and little fingers and generally progresses over time without treatment [1, 2]. The disease is one of the most common hereditary disorders of the connective tissue, and has strong associations with genetic profiles and family history [3]. In previous studies, male gender, diabetes mellitus, excess alcohol consumption and smoking were identified as major risk factors for its development [4, 5]. The current worldwide prevalence of DC is estimated to be around 8.2% [6], a rate which varies greatly with geographic location.

Hand deformity occurs predominantly at the level of the metacarpophalangeal (MCP) and proximal interphalangeal (PIP) joints with subsequent functional disabilities. Patients with DC usually present with a loss of motion due to an extension deficit, that can cause debilitating limitations in daily activities, a reduction of manual ability, and a reduction of quality of life [7].

The most common treatment options are limited fasciectomy (LF), percutaneous needle fasciotomy (NF) and, as a non-surgical alternative, the injection of collagenase clostridium histolyticum (CI). Superiority of one specific treatment modality for DC has yet to be identified or described, and any advantages among LF, NF and CI remain controversial [8, 9]. Previous studies have not been able to demonstrate a relative superiority of different procedures due to insufficient evidence [10] or have not been limited to randomized controlled trials [11].

Hence, the aim of this study was to conduct a comprehensive evaluation of the postoperative outcomes of the different surgical approaches and CI injections for the treatment of DC, to pool the available evidence and provide practitioners with an estimate of what benefits patients may expect from each treatment. To the best of our knowledge, this is the first network meta-analysis performed that assesses treatment outcomes for DC.

## Methods

A thorough systematic and comprehensive literature search was conducted void of language or date restrictions within the PubMed, Cochrane Library and Embase databases according to the Preferred Reporting Items for Systematic Reviews and Meta-Analyses (PRISMA) [12] from inception to August 2021. Index term combinations and MESH search terms used included “dupuytren”, “viking”, “fibroma”, “fascia” and “randomized controlled trial” and comprised all term variations. Citations given in all eligible studies were manually examined and reviewed.

### Selection criteria

We included all randomized controlled trials that reported on the treatment of adult patients (> 18 years) with DC with measurable flexion contracture of the hand. Both primary or recurrent DC were included. Eligible studies had to compare LF, NF and CI with placebo injections or with one another. The outcomes of interest were (a) contracture reduction rates in treated joints (within 0–5° of full extension), (b) relative reduction in total passive extension deficit (TPED), (c) occurrence of one or more adverse events per patient and (d) number of treatment-related adverse events.

The outcomes of interest had to be reported after a follow-up period of at least 1 week. Studies reporting on other treatment modalities and other affected body regions were excluded. Studies that were performed in younger age groups were excluded.

### Literature screening

Upon the database search in the aforementioned databases, search results were imported into Endnote X7 software. Duplicates were identified and excluded. The literature search and evaluation were conducted independently by two researchers (DO and MS) based on predetermined selection criteria. A first selection of studies based on abstracts and titles, was followed by a second evaluation of the full texts for eligibility. During the screening process, inconsistencies were discussed by the two reviewers to reach consensus.

### Data extraction

Data extraction was performed independently by two researchers (DO and MS) and comprised: the first author, publication year, characteristics of the trial, study period, number of total and subgroup patients enrolled, demographics (age, sex, country), disease severity, treatment modalities, preoperative and residual flexion contractures at the joints, CI dosage, primary and secondary outcomes, follow-up duration, occurrence of adverse events according to the terminology applied by the respective authors, and the study results. Conflicting data were discussed and corrected upon agreement.

Complications as defined and reported by the authors were collected for each study arm. To calculate the number of treatment-related adverse events, the number of all as defined adverse events was divided by the highest number possibly attributable to the specific patient cohort for adverse event analyses. To address the overestimation of complication rates in the literature, which reflects the lack of a standardized definition of complications after treatment for DC, we performed additional analyses on adverse events separately for complications that we deemed severe and mostly not attributable to post-procedure complications.

### Assessment of quality

All included studies were randomized controlled trials and their quality was independently evaluated with the Cochrane Collaboration’s tool [13] by two reviewers (DO and MS). The Cochrane Collaboration’s tool allows for the evaluation of risk bias (low, high or unclear) of six particular domains: randomization, possible sources of bias, blinding of subjects and outcome assessors, reporting of incomplete outcome data, allocation concealment and selective outcome reporting.

### Statistical analysis

Traditional pairwise meta-analysis and network meta-analysis of the data from a total of nine included studies was performed using MetaXL (MetaXL, Version 5.3, EpiGear International 2016). MetaXL implements the generalized pairwise modelling (GPM) framework for network meta-analysis, making it more robust compared to other existing methods [14].

For each direct comparison, the weighted mean difference (WMD) in continuous outcomes, the risk difference in dichotomous outcomes, and the mean difference in ratios with 95% confidence intervals (CI) were calculated. The inverse variance heterogeneity (IVhet) model was used as an estimator, as it favors larger trials and produces lower observed variances compared to the random effects model [15]. The studies’ heterogeneity was evaluated with the Cochran’s Q test and the *I *^*2*^ statistic.

To confirm differences across treatment modalities, we also performed a network meta-analysis employing the nodes to represent different interventions and the edges to denote head-to-head comparisons between different treatments. This allowed for indirect comparisons where direct studies were not available for a concluding estimate of effects. Placebo injections were used as the common comparator. Point estimates of the effect sizes were used to rank treatments [16]. The H-statistic was used to assess inconsistencies between direct and indirect evidence. A value of < 3 depicted a minimal, 3–6 a modest and > 6 a gross inconsistency.

## Results

### Literature selection and basic information

**Fig. 1:**
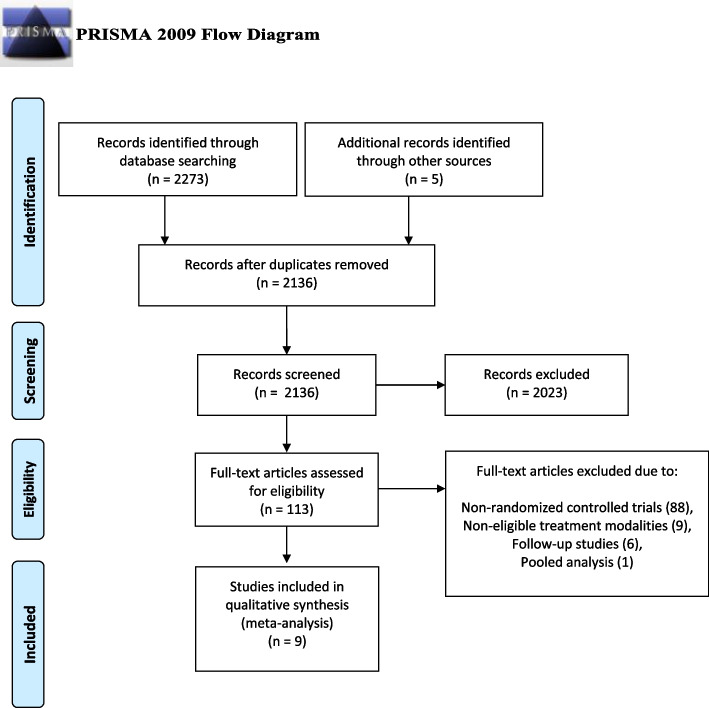
PRISMA Flow Diagram of the study selection process

The literature screening process is portrayed in Fig. 1. A total of 2278 articles were identified during the initial literature search. 152 duplicate entries were recognized and eliminated, resulting in 2136 studies. From these, 2023 articles were excluded after title and abstract evaluation. 104 articles were excluded after full-text screening based on pre-defined exclusion criteria. A total of nine studies were included in the final network meta-analysis (Table 1) [17–25]. The included studies provided data on 903 patients. All patients suffered from flexion contracture of one or more fingers due to DC. Employed treatment modalities included CI versus placebo in four trials, CI versus NF in four trials and LF versus NF in one trial. Table 1 summarizes the details of the nine included studies.

**Table 1:**
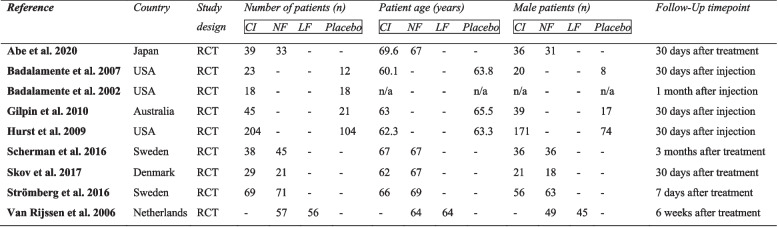
Characteristics of included studies

*CI* Collagenase clostridium histolyticum injection, *NF* Needle fasciotomy, *FSC* Limited fasciectomy, *n/a* not available

The mean age of the patients treated with CI was 64.3 years (± 2.06), with NF 66.8 years (± 1.6), with LF 64 years (± 0) and with placebo 64.2 years (± 0.94). Men accounted for the majority of patients in all groups: 81.5% in the CI group, 86.8% in the NF group, 80.3% in the LF group and 72.3% in the placebo group.

### Quality assessment

**Fig. 2:**
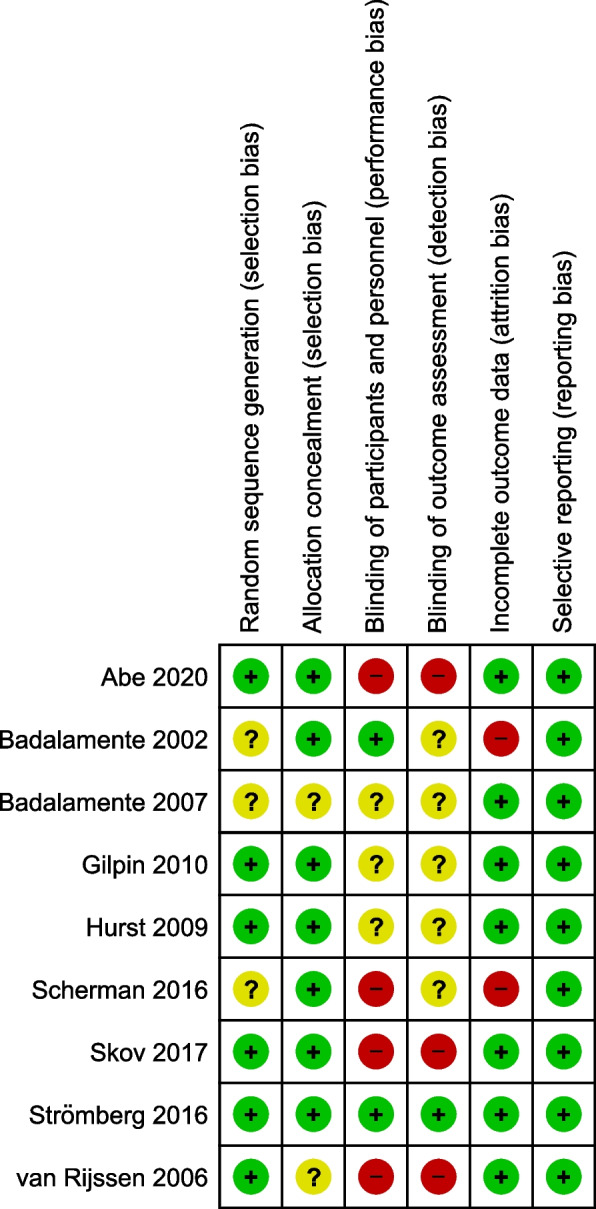
Risk of bias summary

**Fig. 3:**
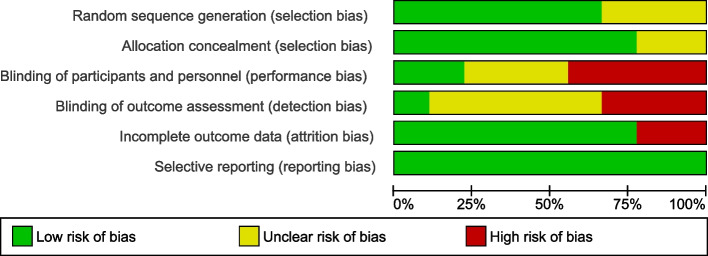
Risk of bias graph

The quality of the studies was assessed using the Cochrane’s Collaboration’s tool. Attrition and selective bias were mostly low for all included studies, except for two studies based on incomplete reporting of adverse events [19, 22]. Allocation concealment was described in the majority of the included studies, except for two [18, 25]. Domain assessment for performance and detection bias revealed a significant bias. Three studies did not allow for complete assessment of random sequence generation due to insufficient information provided [18, 19, 22]. Low total risk of bias seemed to be valid for the majority of included studies, aligning with low risk of bias across the relevant domains. All of the studies included were double-blinded, randomized controlled trials according to our inclusion criteria, however most studies only delivered insufficient information on the methods implied to guarantee blinding of patients and personnel. Figures 2 and 3 demonstrate the results of the risk bias assessment.

### Outcome assessment

**Fig. 4:**
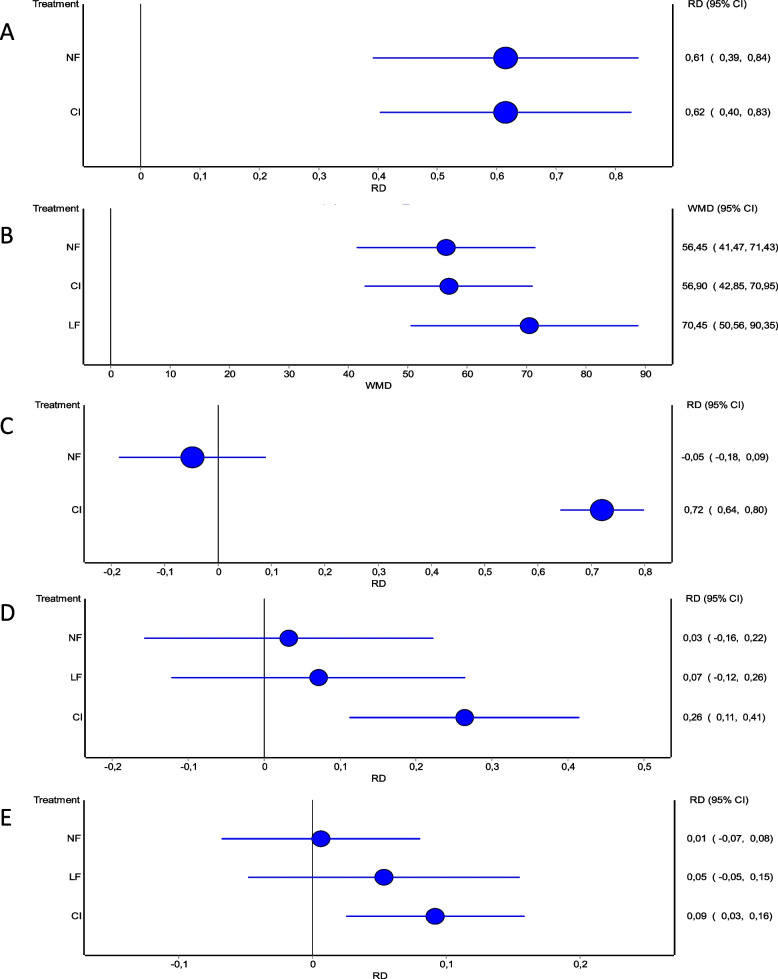
Network meta-analysis of various endpoints. **A** Reduction in primary joint contracture to 0–5° of full extension within 30 days. **B** Relative reduction in total passive extension deficit within 30 days. **C** Occurrence of at least one adverse event per patient. **D** Total number of treatment-related adverse events. **E** Total number of treatment-related severe adverse events

**Fig. 5:**
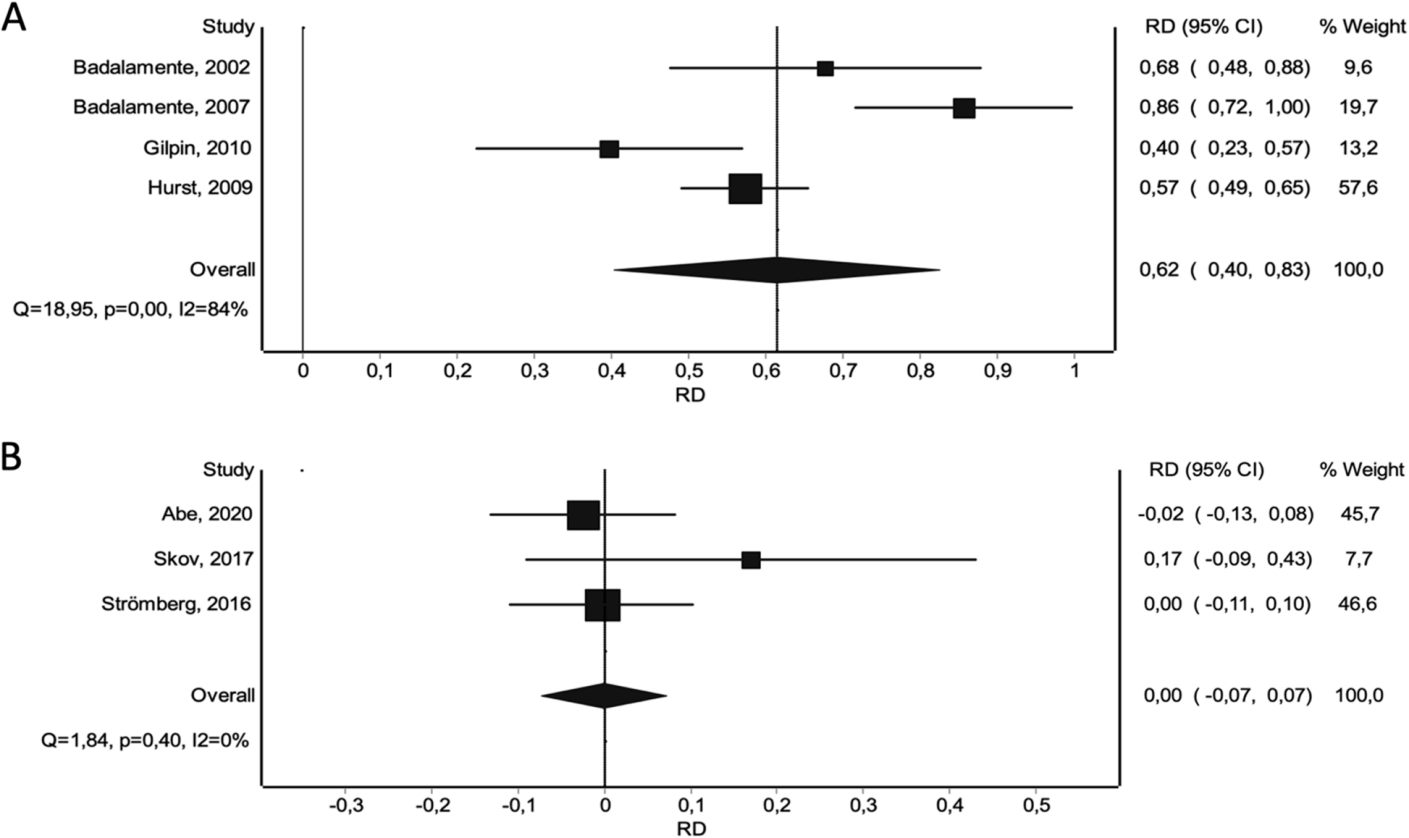
Forest plots of reduction in primary joint contracture to 0–5° of full extension within 30 days. **A** CI versus Placebo. **B** CI versus NF

The results of the network meta-analysis showed that contracture reduction rates in treated joints (within 0–5° of full extension within 30 days) after NF treatment or the last dose of CI were comparable and significantly superior to placebo treatment based on the pooled data (CI vs. placebo: RD 0.62 [0.40 – 0.83]), (NF vs. placebo: RD 0.61 [0.39 – 0.84]). With regard to this outcome endpoint, no significant differences were detected between NF and CI (Fig. 4A). Meta-analysis displays these findings (Fig. 5).

**Fig. 6:**
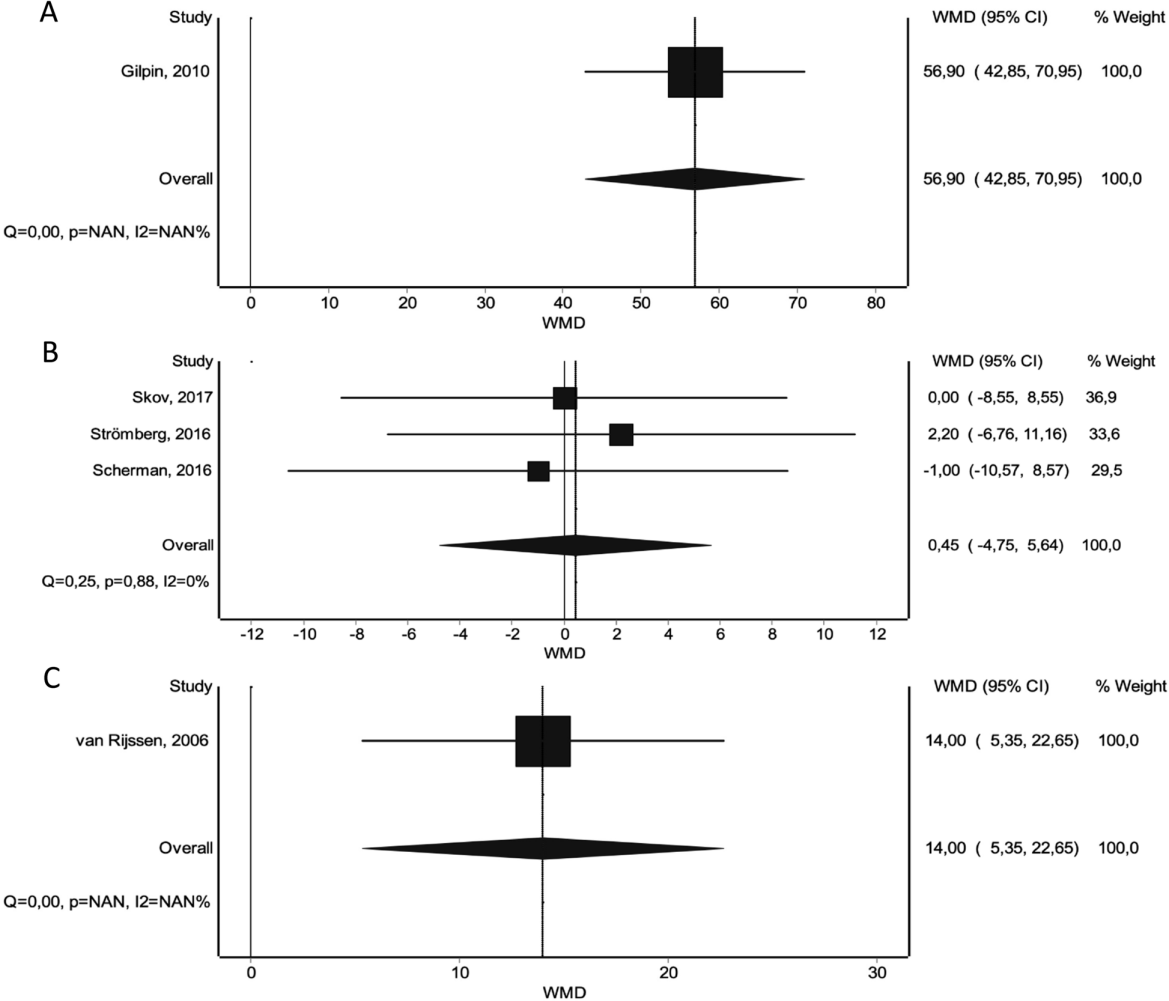
Forest plots of relative reduction in total passive extension deficit within 30 days. **A** CI versus Placebo. **B** CI versus NF. **C** CI versus LF

With regard to post-intervention relative reduction in TPED, all treatment options yielded significantly better results compared to placebo (CI vs. placebo: WMD 56.9 [42.85 – 70.95]), (NF vs. placebo: WMD 56.45 [41.47 – 71.43]), (LF vs. placebo: WMD 70.45 [50.56 – 90.35]) (Fig. 6). LF showed significantly superior reduction rates compared to CI and NF (Fig. 4B).

A comparative and quantitative description of all reported adverse events in the included studies is presented in Table 2. The reported complications rates for respective adverse events varied from 0 to 100%. There were significant inconsistencies in complication reports and accounts of adverse events were void of a general standardized definition across the studies. We found 31 different accounts of adverse events reported in the included studies of which 18 were considered severe and most likely non-related to procedural effects after consensus, e.g. tendon and nerve injury, distal ischemia, lymphangitis, rash or myalgia (see Table 2).

**Table 2:**
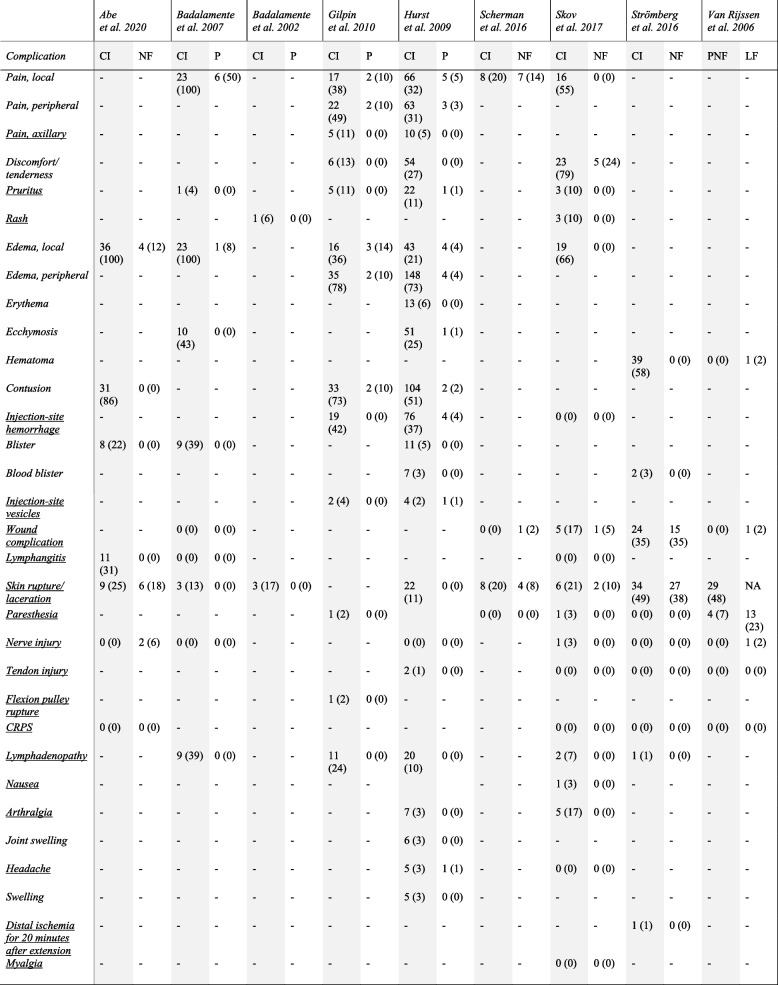
Adverse events reported in the included studies. The percentage for the study arms is in parentheses. Underlined complications were considered severe

**Fig. 7:**
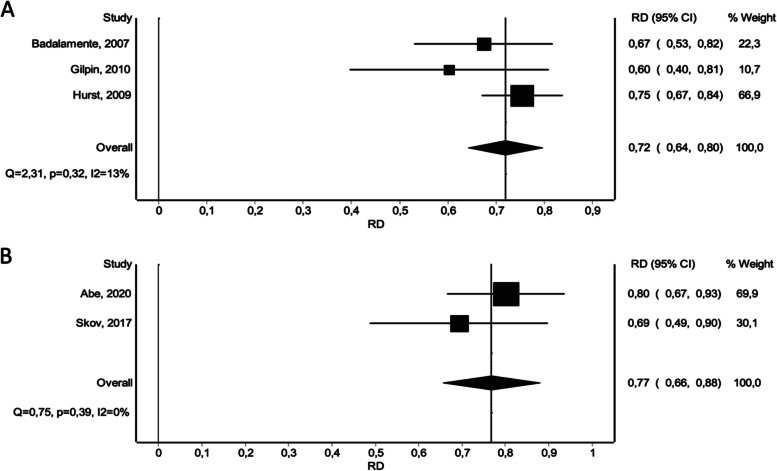
Forest plots of occurrence of at least one adverse event per patient. **A** CI versus Placebo. **B** CI versus NF

Assessment of the occurrence of one or more adverse events following treatment revealed a greater risk for patients treated with CI and a reduced risk for patients treated with NF compared to placebo (CI vs. placebo: RD 0.72 [0.64 – 0.80]), (NF vs. placebo: RD -0.04 [-0.18 – 0.09]). NF was found to be associated with a significantly reduced risk of occurrence of one or more adverse events (Fig. 4C). This is in accordance with the results of the meta-analysis (Fig. 7).

**Fig. 8:**
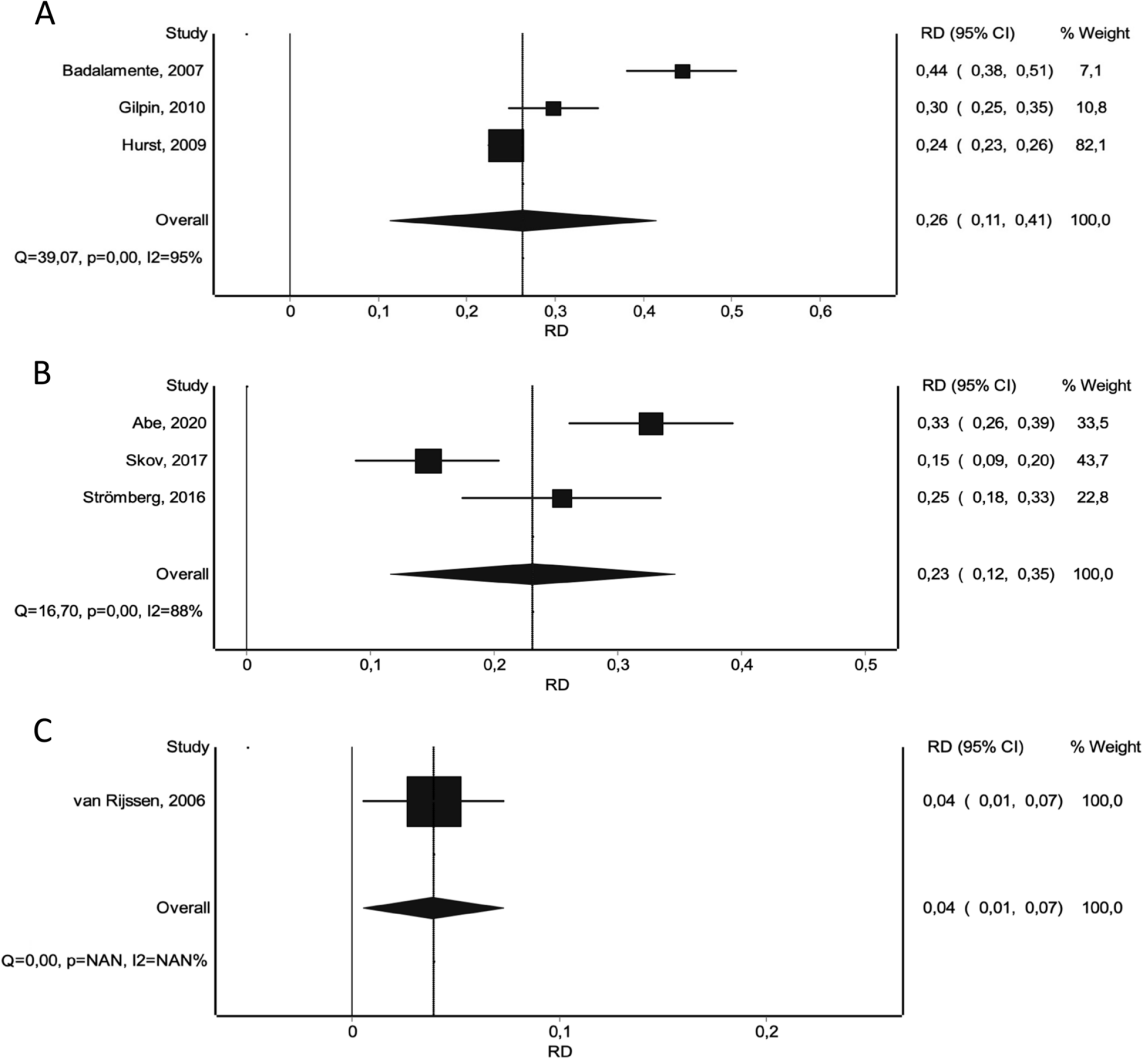
Forest plots of number of treatment-related adverse events. **A** CI versus Placebo. **B** CI versus NF. **C** CI versus LF

**Fig. 9:**
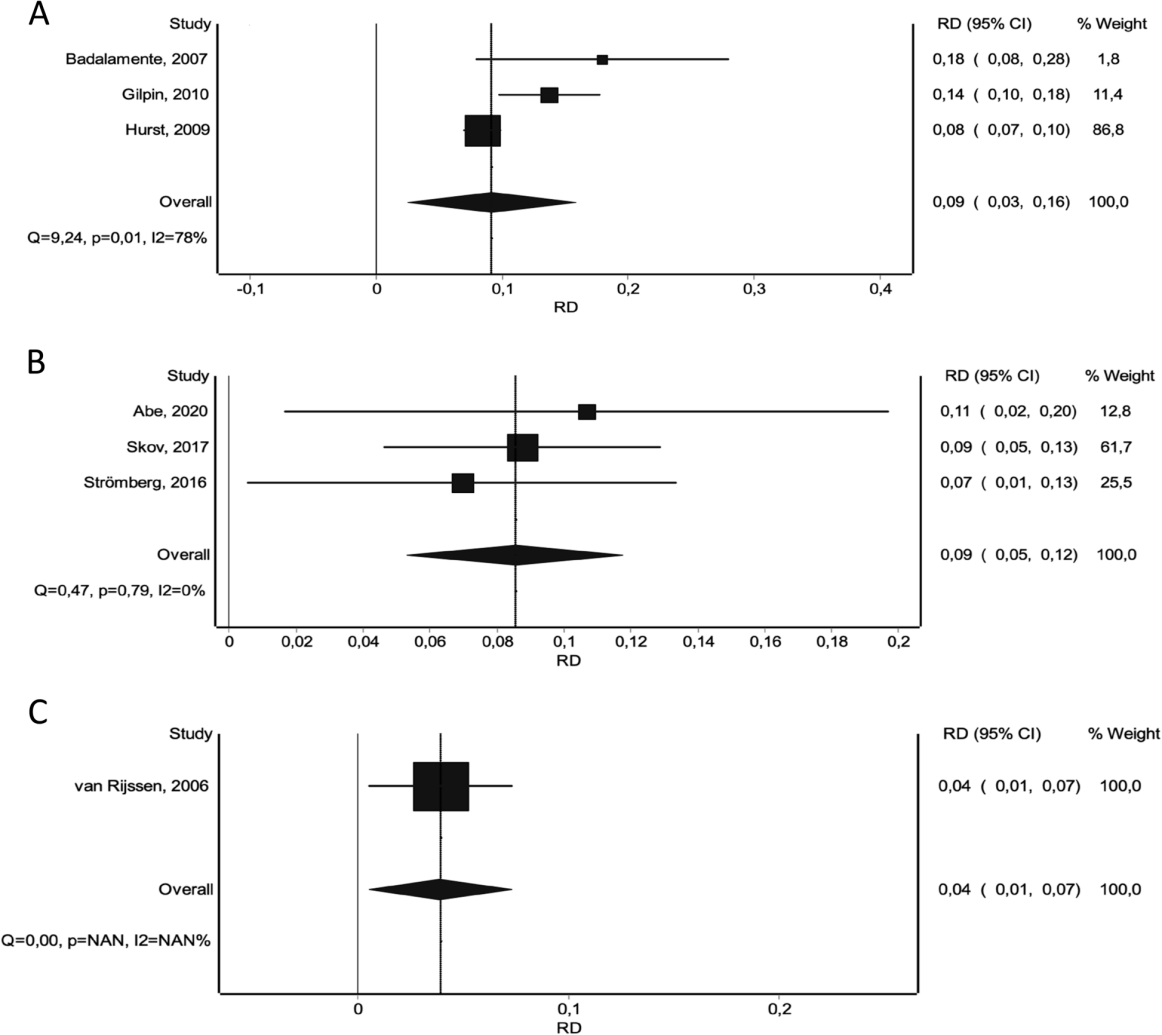
Forest plots of number of severe treatment-related adverse events. **A** CI versus Placebo. **B** CI versus NF. **C** CI versus LF

Similarly, the number of treatment-related adverse events appeared to be significantly higher for CI, NF and LF compared to placebo, respectively: (CI vs. placebo: RD 0.26 [0.11 – 0.41]), (NF vs. placebo: RD 0.03 [-0.16 – 0.22]), (LF vs. placebo: RD 0.07 [0.12 – 0.26]). CI did result in significantly more adverse events, compared to NF and LF (Fig. 8). NF was associated with a lower occurrence of adverse events compared to LF, however this result showed no statistical significance (Fig. 4D). When omitting the non-severe adverse events, the overall effect stayed persistent, however we noted a significant overall risk difference, a reduced occurrence of adverse events for CI and NF and an increase of adverse events for LF compared to placebo, respectively: (CI vs. placebo: RD 0.09 [0.03 – 0.16]), (NF vs. placebo: RD 0.01 [-0.07 – 0.08]), (LF vs. placebo: RD 0.05 [-0.05 – 0.15]). Again, CI did result in significantly more adverse events, compared to NF and LF (Fig. 9) and similarly NF showed a lower occurrence of adverse events compared to LF, again with no statistical significance (Fig. 4E).

## Discussion

As there is currently no cure for DC, the primary aim of treatment continues to be the excision, dissolvement or disruption of the fibrous cords that prohibit finger extension with the intention of releasing finger contracture. Several treatment options have emerged, with non-operative options ranging from physical therapy, radiotherapy to extracorporeal shockwave treatment. A fairly new treatment option is the injection of the collagenase produced by *Clostridium histolyticum*. Its treatment effect is based on the degradation of collagen fibers within the DC cords which are ultimately disrupted by forced extension of the injected finger the following day. Food and Drug Administration (FDA) approval in the United States took place in 2010 [26]. The interim treatment license in the European Union, that had been introduced in March 2011, was revoked by the manufacturer in 2019 [27].

The mainstay of treatment for advanced DC is surgery, with several options available of different levels of invasiveness. Among these, NF or aponeurotomy, segmental aponeurectomy and LF, as the most popular treatments, present adequate options.

Although there are multiple treatment options, a paucity of RCTs in the literature persists. Previous studies have, thus, not been able to demonstrate a relative superiority of any of the different treatment procedures due to insufficient high-quality evidence.

In this network meta-analysis, we show that CI presents an effective treatment option, however it does not yield superior short-term results compared to NF despite a higher complication profile. Although CI, rather than NF, is thought to be associated with greater functional improvement in some reports [28], in the available RCTs the differences between the treatment modalities appeared trivial when looking at TPED and contracture reduction to 0°—5° of full extension in treated joints within 30 days. Our results are in line with recent trials that have shown similar short- to medium-term functional results [14].

Similarly, the available literature is conflicted with regard to the role of LF, with some studies showing varying results in favor of CI when evaluating PIP and MCP joint contractures [29, 30], whereas others demonstrated a better performance of LF at early stages and 2-years postoperatively in PIP joints as well as in MCP joints [31].

In our study, LF seemed to be promising compared to NF and CI with regard to relative reduction of TPED and showed an adequate safety profile compared to CI, however only one study contributed to the weighted effect in the analysis [25]. Based on the data presented, the limited follow-up periods and given that recurrence and revision rates have not been included in the analysis, no conclusions can be drawn on any long-term effects. It can only be assumed, that CI and NF, demonstrably less invasive surgical interventions, do not achieve a long-lasting effect comparable with that attained by LF.

Additionally, compared with the other procedures, CI was associated with an increased risk of adverse event occurrence, as well as a higher total number of adverse events. Generally, patients with severe or recurrent DC are more likely to experience complications [32]. In light of this, patient education is paramount as an increase in potential postoperative complications seems to be attributable to the procedure selected. Our results are at odds with previous studies that have shown similar risk and side-effect outcomes of CI in comparison with LF [29, 33]. Sanjuan-Cerveró et al. [34] have previously shown in their meta-analysis of ten cohort studies with random-effects modelling that patients treated with CI had increased odds of adverse effects compared with those treated by LF, which agreed with our analysis. However, their statistical difference disappeared when mild adverse effects were removed from their evaluation.

Overall, the design quality and reporting of studies in DC surgery remains generally poor [10]. As the pressure to warrant the cost-effectiveness of therapeutic measures in the healthcare sector persists, future research should aim towards high-quality comparative analyses of different procedures with validated and reliable outcome assessment tools.

This network meta-analysis was based solely on RCTs and treatment effects were evaluated in a network using a generalized pairwise modelling (GPM) framework. The overall risk of bias assessment identified low to moderate risk profiles. Despite its strengths, it has several limitations. Only nine studies were found to be eligible for final analysis, all of which provided short follow-up data. Moreover, treatment effect evaluation for LF was based on one study [25]. Hence, our results have to be interpreted with caution given that only a single LF study added weight to the analysis, which may have been a major study bias. Similarly, long-term functional treatment effects cannot be extrapolated from this analysis given the limited follow-up time of the underlying studies. The assessment of severe adverse events was determined in consensus; however this may not reflect a general evaluation. Due to potential attrition bias in two of the original studies the analysis was further limited.

Taken all together, the data from the included RCTs support the short-term efficacy of all assessed treatment modalities in DC. However, indications for the superiority of one specific intervention are limited. Based on published trials, the selection of the most suitable treatment modality remains clinically important in order to guarantee optimal functional results with few adverse events. For this, large-scale prospective trials comparing different treatment modalities for DC should be conducted over the course of long follow-up periods. In line with previous reports [34, 35], we suggest the consensus-based generation of a standardized classification to rank complications following DC treatment in a reproducible and objective manner. This would allow for a reduction in interobserver variability in the grading of complications and thereby enable a more sound assessment of treatment effects.

## Conclusion

In conclusion, our data suggest that LF yields promising short-term functional results in terms of relative reduction of TPED when compared to CI and NF and may be able to provide patients with severe disease superior flexion contracture release postoperatively. Still, our results have to be interpreted with caution given the limited evidence for LF in our analysis. CI yielded similar results regarding reduction of primary joint contracture compared to NF and presented a valid treatment alternative, however it was associated with a higher risk of overall and severe adverse effects. Given these results, it is unfortunate that the manufacturer’s withdrawal of CI is still in effect in the European Union, denying a valid treatment option to a large patient cohort in which surgical treatment may not be the best option.

## Data Availability

The datasets used and analyzed during the current study are available from the corresponding author on reasonable request.

## Abbreviations

CI: Collagenase clostridium histolyticum injections
DC: Dupuytren’s contracture
FDA: Food and Drug Administration
GPM: Generalized pairwise modelling
LF: Limited fasciectomy
MCP: Metacarpophalangeal
NF: Needle fasciotomy
PIP: Proximal interphalangeal
RCT: Randomized controlled trial
RD: Risk difference
TPED: Total passive extension deficit
WMD: Weighted Mean Difference
